# Correlation between serum microRNA-122 and VEGF expression and pregnancy outcome in gestational diabetes mellitus patients

**DOI:** 10.12669/pjms.40.3.8467

**Published:** 2024

**Authors:** Hongmei He, Yi Wang, Haijiao Wang, Yulan Ma, Pan Zhang

**Affiliations:** 1 Hongmei He, Department of Clinical Laboratory, The Fourth Hospital of Shijiazhuang, Shijiazhuang, Hebei 050000, China; 2 Yi Wang, Department of Clinical Laboratory, The Fourth Hospital of Shijiazhuang, Shijiazhuang, Hebei 050000, China; 3 Haijiao Wang, Department of Clinical Laboratory, The Fourth Hospital of Shijiazhuang, Shijiazhuang, Hebei 050000, China; 4 Yulan Ma, Department of Clinical Laboratory, The Fourth Hospital of Shijiazhuang, Shijiazhuang, Hebei 050000, China; 5 Pan Zhang, Department of Clinical Laboratory, The Fourth Hospital of Shijiazhuang, Shijiazhuang, Hebei 050000, China

**Keywords:** Gestational diabetes mellitus, miR-122, VEGF, Pregnancy outcome

## Abstract

**Objective:**

Gestational diabetes mellitus (GDM) seriously influences the health of mothers and babies, and there are still no effective early diagnostic markers. Therefore, our study planned to probe the correlation between serum microRNA-122 and VEGF expression and pregnancy outcome in GDM patients.

**Methods:**

This was a retrospective study of the correlation between serum microRNA-122 and vascular endothelial growth factor (VEGF) expression and pregnancy outcome in GDM patients. Sixty GDM patients admitted to the Fourth Hospital of Shijiazhuang from January 2021 to October 2022 were included in the research group (RG), and another 60 healthy pregnant women were included in the control group (CG). Serum miR-122 and VEGF levels were quantified using quantitative real-time polymerase chain reaction. The value of miR-122 and VEGF in predicting adverse pregnancy outcomes was analyzed by receiver operating characteristic curve.

**Results:**

Serum miR-122 and VEGF levels in the RG were higher relative to the CG. The total occurrence of adverse pregnancy outcomes in the RG was higher relative to the CG (P<0.05). Serum miR-122 together with VEGF levels in the poor outcome group was higher relative to the good outcome group (P<0.05). ROC analysis revealed that miR-122 and VEGF could be used to predict adverse pregnancy outcome (P<0.0001). The area under the curve of miR-122 was 0.860, 95% confidence interval (CI) =0.793-0.926, and the area under the curve of VEGF was 0.780, 95% CI =0.694-0.866. Serum levels of miR-122, VEGF were positively related with abortion, preterm delivery, low birth weight infants, macrogenesis infants, and fetal development abnormalities (P<0.001).

**Conclusion:**

The higher serum miR-122 and VEGF levels in GDM patients with satisfactory blood glucose control, the greater the probability of adverse pregnancy outcome, which should be paid attention to by clinicians.

## INTRODUCTION

Gestational diabetes mellitus (GDM) belongs to aberrant glucose tolerance that first occurs or is discovered during pregnancy.^1^ GDM can cause the dysfunction of sugar, fat and protein metabolism in pregnant women, resulting in reduced glucose uptake and utilization, reduced protein decomposition, and increased lipolysis and oxidation, which is easy to cause amniotic fluid excess, spontaneous abortion, deformity, macrosomia, pregnant women ketoacidosis and other diseases, which is more harmful to mother and child.^2^ At present, the incidence of GDM in China ranges from 17.5% to 18.9%.^3^ The pathogenesis underlying GDM is related to insulin resistance and insufficient insulin secretion.^4^ Dietary intervention, exercise therapy and insulin therapy are used to control the blood glucose level of patients with GDM to reduce the risk of pregnancy complications and promote benign pregnancy outcomes.^5^,^6^ Glucose tolerance tests are often used to diagnose GDM, but this method cannot predict the risk of disease, and can only be diagnosed when the disease appears.^7^ Therefore, early estimation of the risk of adverse pregnancy outcomes in GDM patients and timely implementation of reasonable intervention are of great significance for promoting benign prognosis.

Epigenetic regulation has a crucial potential in the pathogenesis of GDM.^8^ The epigenetic regulation contains histone modification, DNA methylation along with microRNA (miRNA) depletion, which are strongly associated with each other and affect protein synthesis patterns.^9^ MiRNAs belong to highly conserved small noncoding RNAs and affect the progression of diseases via post-transcriptionally modulating gene expression.^10^ As reported previously, many miRNAs such as miR-20a, miR-30d, and miR-125b, have functioned as promising diagnostic as well as therapeutic methods because of their relation with GDM.^11^ As reported previously, miR-122 is involved in the progression of various diseases, such as liver disease,^12^ cervical cancer,^13^ and cardiovascular fibrosis and related diseases,^14^ which can function as a potential specific biomarker or the potential therapeutic target of diseases. However, the expression and role of miR-122 in GDM patients are unclear.

Vascular endothelial growth factor (VEGF) is involved in the pathogenesis underlying diabetes mainly via promoting vascular permeability, altering the gene expression in vascular endothelial cells, elevating the mitosis of genes, and inducing new angiogenesis.^15^ In addition, VEGF plays a critical role in vascular changes of GDM.^16^ For example, Troncoso F et al. point that GDM is associated with increased pro-migratory activation of VEGF receptor-2 and reduced expression of VEGF receptor 1.^17^ Sundar Krishnasamy et al. discover that VEGF165b/VEGFTOTAL ratio plays an important role in GDM in association with vascular inflammation.^18^ In this study, we probed the correlation between serum miR-122 and VEGF expression and pregnancy outcome in GDM patients.

## METHODS

This was a retrospective study of the correlation between serum microRNA-122 and VEGF expression and pregnancy outcome in GDM patients. A total of 60 GDM patients admitted to the Fourth Hospital of Shijiazhuang from January 2021 to October 2022 were included in the research group (RG), among them, there were 42 cases of primipara and 18 cases of meningopara. The average age was 29.38±3.12 years, ranging from 21 to 38 years. The average gestational age was 35.25±3.36 weeks, ranging from 32 to 39 weeks. At the same time, 60 healthy pregnant women were included in the control group (CG), among them, 43 cases of primipara and 17 cases of meningopara. The average age was 29.33±3.07 years, ranging from 20 to 37 years. The average gestational age was 35.27±3.18 weeks, ranging from 31 to 39 weeks. No difference was discovered in general data of patients between both groups (P>0.05), indicating comparable. Peripheral venous blood of the subjects was collected, and these patients were informed and have signed the informed consents. This study was approved by the Fourth Hospital of Shijiazhuang under the approval number of 20190047.

### Inclusion criteria:

- Patients in the RG met the diagnostic criteria for GDM.
- All subjects gave their informed consent.

### Exclusion criteria:

- Multiple pregnancies.
- Suffering from cardiovascular, liver and kidney diseases before pregnancy
- Patients with polycystic ovarian syndrome and malignant tumor.
- Patients with acute and chronic infections, immune system diseases, mental system diseases

### Serum VEGF level detection

Venous blood of the subjects was collected for 3-4 mL, centrifuged for 5 min at a rotating speed of 4800 r/min, and VEGF was determined by enzyme-linked immunosorbent assay using IAMMGE specific protein analyzer (Beckman, USA).

### Serum miR-122 level detection using quantitative real-time polymerase chain reaction (qRT-PCR):

A total of 3-4 mL of peripheral venous blood of the subjects was collected, centrifuged, and the serum was collected, placed in a coagulation tube, mixed with miRNA extractor and divided into an eppendorf (EP) tube, and placed in a -70°C refrigerator for freezing storage. miRNA was extracted, samples were taken out and placed at room temperature, nucleic acid and nuclear protein were completely separated, chloroform was added for 0.2 mL, shocked and centrifuged, the upper water phase was absorbed and placed in a new EP tube, 1/3 times anhydrous ethanol was added to mix, and then added to the adsorption column. After centrifugation, the flowing liquid was gathered into a new EP tube, 2/3 times anhydrous ethanol was added and mixed well, all the solution was added into the adsorption column with a liquid transfer device, centrifugation at 7500 r/min for five minutes, and the waste liquid was dumped in the collection pipe. Finally, the adsorption column was placed back into the collection pipe, centrifuged for two minutes at 12000 r/min, placed into a new EP tube, treated with 30 μL RNase-free water. The adsorption column was abandoned, and the effluent was retained. The cDNA was synthesized by reverse transcription, and various reaction components were added into a centrifuge tube, mixed with 12000 r/min, and centrifuged for 3-5 s. The reaction mixture was treated with a TPfofessional Standard PCR instrument (Biometra, Germany) to inactivate the enzyme by taking a warm bath at 37°C for 60 minutes and heating at 85°C for five minutes. qRT-PCR reaction of the primer sequences miR-122: forward: 5’-GCGGTCGACATGGTGGAATGTGGAGGTGAAG-3’, reverse: 5’-GGAATTCAAAAAAGATTGAGAAGACTGATATC-3’. U6: forward: 5’- CTCGCTTCGGCAGCCACA-3’, reverse: 5’-AACGCTTCACGAATTTGCGT-3’. After obtaining cDNA samples, the corresponding samples and reagents were added, and real-time fluorescence quantitative PCR instrument was used for reaction system operation. 2^-ΔΔCt^ method was used for data acquisition.

### Observation indexes:

(1) Levels of miR-122 and VEGF were detected in both groups.
(2) The occurrence of adverse pregnancy outcomes containing miscarriage, premature delivery, low birth weight infants, macrosomia, and fetal dysplasia was compared between both groups.
(3) Serum levels of miR-122 and VEGF in different pregnancy outcomes were compared in the RG.
(4) The value of miR-122 and VEGF in predicting adverse pregnancy outcomes was analyzed.
(5) The correlation between miR-122 and VEGF and adverse pregnancy outcomes was assessed.

### Statistical analysis

SPSS 22.0 statistical software was implemented for data analysis, measurement data were expressed as mean and standard deviation, and t test was implemented for comparison. The statistical data were represented by the number of cases and percentage [n (%)], and χ^2^ test was implemented for comparison. The value of miR-122 and VEGF in predicting adverse pregnancy outcome was analyzed by receiver operating characteristic (ROC) curve, and the area under curve (AUC) is defined as the area under the ROC curve surrounding the axis. The value of AUC >0.5 indicates significance. In the case of AUC >0.5, the closer the value of AUC is to 1, the better the diagnostic effect is. P<0.05 was significant.

## RESULTS

### Serum miR-122 and VEGF levels in both groups

**Fig.1:**
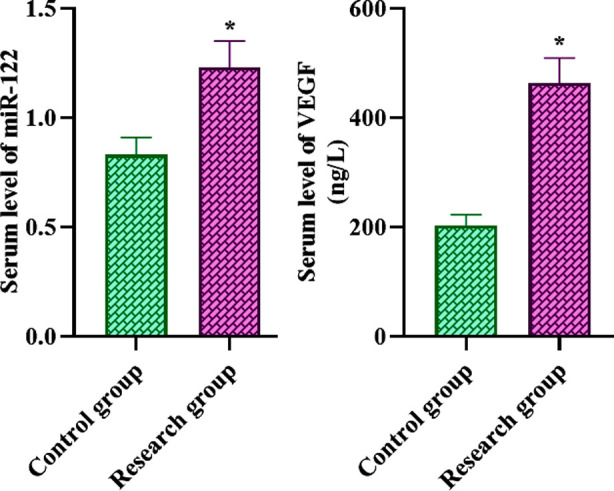
Serum miR-122 and VEGF levels in both groups. *P<0.05.

Serum miR-122 and VEGF levels of patients in the RG were higher relative to the CG (P<0.05, Fig.1).

### Incidence of adverse pregnancy outcomes in both groups

The total incidence of adverse pregnancy outcomes in the CG was 8.33%, which was lower than 21.67% in the RG (P<0.05, Table-I).

**Table-I:** Incidence of adverse pregnancy outcomes in both groups.

Groups	N	Miscarriage	Premature delivery	Low birth weight infants	Macrosomia	Fetal dysplasia	Total incidence rate (%)
Control group	60	4	2	2	2	3	13 (21.67%)
Research group	60	1	1	2	0	1	5 (8.33%)
*χ* ^2^		4.18					
*P*		<0.05					

### Serum miR-122 and VEGF levels in patients with different pregnancy outcomes in the research group

**Fig.2:**
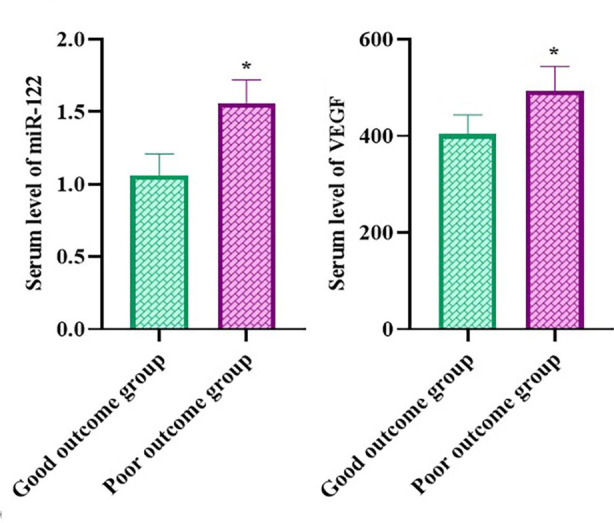
Serum miR-122 and VEGF levels in patients with different pregnancy outcomes in the research group. *P<0.05.

The serum miR-122 and VEGF levels of patients in the poor outcome group were elevated relative to the good outcome group (P<0.05, Fig.2).

### ROC analysis of miR-122 and VEGF in predicting adverse pregnancy outcomes

**Fig.3:**
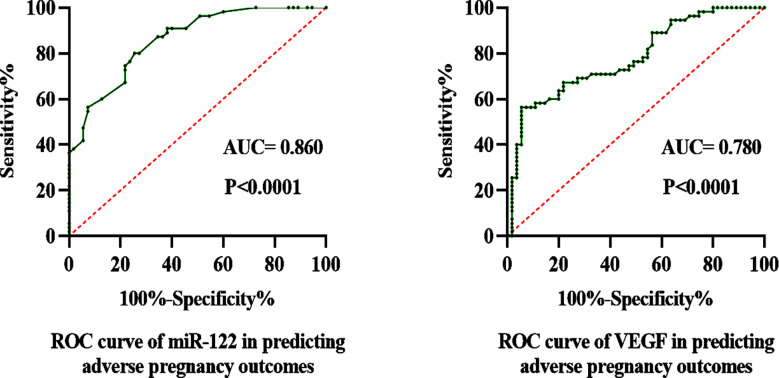
ROC analysis of miR-122 and VEGF in predicting adverse pregnancy outcomes.

ROC analysis showed that miR-122 and VEGF could be used to predict adverse pregnancy outcome (P<0.0001). The area under the curve of miR-122 was 0.860, 95% CI = 0.793-0.926, and the area under VEGF curve was 0.780, 95% CI = 0.694-0.866 (Fig.3).

### Correlation analysis of miR-122, VEGF and adverse pregnancy outcomes

Serum levels of miR-122, VEGF were positively correlated with abortion, preterm delivery, low birth weight infants, macrogenesis infants, and fetal development abnormalities (P<0.001, Table-II).

**Table-II:** Correlation analysis of miR-122, VEGF and adverse pregnancy outcomes.

Indexes	Miscarriage		Premature delivery		Low birth weight infants		Macrosomia		Fetal dysplasia	

	r	P	r	P	r	P	r	P	r	P
miR-122	0.635	<0.001	0.563	<0.001	0.654	<0.001	0.589	<0.001	0.645	<0.001
VEGF	0.615	<0.001	0.548	<0.001	0.647	<0.001	0.576	<0.001	0.631	<0.001

## DISCUSSION

The study revealed the higher levels of serum miR-122 and VEGF in GDM patients and demonstrated that they were associated with adverse pregnancy outcome. The role of miR-122 in GDM has been studied, but also includes controversial findings. Ye Z et al. demonstrate that miR-122 expression is lower in plasma exosomes of women with GDM.^19^ Dinesen S et al. hold the opinion that miR-122 has no significant correlation with GDM through meta-analysis.^20^ Virginie Gillet reveals that miR-122 is upregulated in the blood extracellular vesicles of GDM patients^21^, which is consistent with our study. As for VEGF, it is widely present in placenta and blood of pregnancies complicated with GDM.^17^,^22^,^23^

Pathological changes of blood glucose can affect many morphological changes, such as placental cytotrophoblast hyperplasia, villi arteriole hyperplasia, small vessel lumen stenosis, and thickening of the trophoblast basement membrane.^24^ At the same time, a series of changes such as villous capillary overfilling and telangiectasia are the main causes of chronic placental hypoxia, neonatal asphyxia, premature rupture of membranes, fetal distress, as well as other adverse neonatal outcomes.^25^ In addition, elevated blood sugar can increase the amount of cholic acid, cause intrahepatic cholestasis, increase uterine prostaglandin levels, stimulate uterine smooth muscle oxytocin receptors, cause severe contractions, and result in premature birth.^26^ Therefore, early and timely diagnosis and treatment of GDM are very important to improve maternal and infant outcomes.^27^,^28^

The internal environment of placental trophoblast cells of GDM is destroyed, and the cell function is adaptive, which can induce the apoptosis of placental tissue.^29^ This process is closely linked to the blood supply of placental tissue, and VEGF factor is one of the strongest known angiogenic factors.^30^ The outcomes of this study revealed that the level of VEGF in the RG was higher relative to the CG, and it was preliminarily speculated that the abnormal expression of serum VEGF might be related to GDM. The reasons may be:(1) Dense placental blood vessels and vascular endothelial injury in the placental bed may lead to blood perfusion insufficiency and imbalance of vasoactive substance secretion, and eventually lead to loss of oxygen supply in the placenta.^31^ (2) In the condition of hyperglycemia, cellular anaerobic colysis is enhanced, and to adapt to the high glucose environment, the transcription level of glycolytic-related enzyme genes will be changed, leading to chronic hypoxia of placental microvessels.^32^ Hence, abnormal increase of VEGF expression may lead to placental disturbance, which is an important cause of premature delivery and abortion. In line with our outcomes, it has been documented that pregnancies complicated by GDM are characterized by increased placental expression of VEGF.^22^

Nowadays, the role of miRNA in GDM is attracting more and more attention.^33^ For example, miRNA-221 inhibits islet β cell function in GDM by regulating p21-activated protein kinase (PAK1).^34^ MiR-351 relieves insulin resistance by the phosphatidylinositol-4,5-bisphosphate 3-kinase/protein kinase B (PI3K/AKT) pathway in GDM mice.^35^ Herein, the results of our study unveiled that the serum miR-122 expression in the RG was higher than that in the CG, suggesting that the abnormal expression of miR-122 in GDM patients may be related to insulin resistance. Consistent with our findings, Abnoos Mokhtari Ardekani et al. indicate that miR-122 expression level in type 2 diabetes mellitus (T2DM) patients is significantly higher than that in control subjects.^36^ Moreover, miR-122 is a potential novel tool for the early diagnosis and risk estimation of non-alcoholic fatty liver disease in T2DM patients.^37^

In addition, the outcomes of this study demonstrated that the total incidence of adverse pregnancy outcomes in the RG was significantly higher than the CG, and the levels of miR-122 and VEGF in the poor outcome group were elevated relative to the good outcome group. Meanwhile, ROC analysis uncovered that miR-122 and VEGF could be used to predict adverse pregnancy outcomes, suggesting that high levels of miR-122 and VEGF were significantly related to adverse pregnancy outcomes. Finally, correlation analysis showed that miR-122 and VEGF were positively correlated with abortion, premature delivery, low birth weight infants, macrosomia, and fetal development abnormalities, which were basically similar to previous studies.^38^ Therefore, the above indicators should be paid close attention in the subsequent diagnosis and treatment of GDM patients.

### Limitations

The sample size is not large enough. Expression levels of miR-122 and VEGF in placenta tissues of GDM patients remain unknown. We lack an immuno-histochemistry testing of VEGF and an in-Situ Hybridization testing of miR-122 to further reveal the association of placental miR-122 and VEGF expression with the adverse pregnancy outcomes.

## CONCLUSION

The higher the levels of serum miR-122 and VEGF in GDM patients with satisfactory blood glucose control, the greater the probability of adverse pregnancy outcome, which should be paid attention to by clinicians.

### Author`s contributions:

**HH** wrote the initial manuscript and performed assays.

**YW** designed, supervised the study, and revised the manuscript.

**HW** collected data and performed data analysis.

**YM** performed data analysis and prepared tables.

**PZ** prepared figures.

All authors are responsible and accountable for the accuracy of the work.
